# Social Determinants of Health and US Health Care Expenditures by Insurer

**DOI:** 10.1001/jamanetworkopen.2024.40467

**Published:** 2024-10-23

**Authors:** Giridhar Mohan, Darrell J. Gaskin

**Affiliations:** 1 Bloomberg School of Public Health, Johns Hopkins University, Baltimore, Maryland; 2 Department of Health Policy and Management, Bloomberg School of Public Health, Johns Hopkins University, Baltimore, Maryland

## Abstract

**Importance:**

US health expenditures have been growing at an unsustainable rate, while health inequities and poor outcomes persist. Targeting social determinants of health (SDOH) may contribute to identifying and controlling health care expenditures.

**Objective:**

To determine whether SDOH are associated with US health care expenditures by Medicare, Medicaid, and private insurers.

**Design, Setting, and Participants:**

Cross-sectional study of adults, representing the US civilian, noninstitutionalized population with Medicare, Medicaid, or private coverage, from the 2021 Medical Expenditure Panel SDOH Survey. Data analysis was conducted from October 2023 to April 2024.

**Exposure:**

SDOH as individual-level, health-related social needs categorized by Healthy People 2030 domains: (1) educational access and quality, (2) health care access and quality, (3) neighborhood and built environment,(4) economic stability, and (5) social and community context.

**Main Outcomes and Measures:**

The primary outcome was health care expenditures (US dollars) by Medicare, Medicaid, and private insurers. A 2-part econometric model (probit regression model and generalized linear model with gamma distribution) was used.

**Results:**

Among the 14 918 insured adults in the analytic sample (mean [SD] age, 52.5 [17.9] years; 8471 female [56.8%]), the majority had middle to high family income (10 524 participants [70.5%]) and were privately insured (10 227 participants [68.5%]). Annual median (IQR) expenditure was $1648 ($389-$7126) for Medicaid, $3643 ($1321-$10 519) for Medicare, and $1369 ($456-$4078) for private insurers. Educational attainment and social isolation were associated with Medicaid expenditures. Medicaid beneficiaries with a high school diploma or general educational development certificate had on average (mean difference) $2245.39 lower annual Medicaid expenditures (95% CI, −$3700.97 to −$789.80) compared with beneficiaries with less than high school attainment. Compared with those who never felt isolated, Medicaid beneficiaries who often felt isolated had on average $2706.94 (95% CI, $1339.06-$4074.82) higher annual Medicaid expenditures. Health care access, built environment, and economic stability were associated with Medicare expenditures. Medicare beneficiaries living in neighborhoods with lower availability of parks had on average $5959.27 (95% CI, $1679.99 to $10 238.55) higher annual Medicare expenditures. Medicare beneficiaries who were very confident in covering unexpected expenses had on average $3743.98 lower annual Medicare expenditures (95% CI, −$6500.68 to −$987.28) compared with those who were not confident. Medical discrimination and economic stability were associated with private expenditures. Private insurance beneficiaries who experienced medical discrimination had on average $2599.93 (95% CI, $863.71-$4336.15) higher annual private expenditures compared with those who did not. Private beneficiaries who were contacted by debt collections in the past year had on average $2033.34 (95% CI, $896.82 to $3169.86) higher annual private expenditures compared with those who were not contacted.

**Conclusions and Relevance:**

In this cross-sectional study of 14 918 insured adults, individual-level SDOH were significantly associated with US health care expenditures by Medicare, Medicaid, and private insurers. These findings may inform health insurers and policymakers to incorporate SDOH in their decision-making practices to identify and control health care expenditures, advancing health equity.

## Key Points

**Question:**

Are social determinants of health (SDOH) among US insured adults associated with health care expenditures by Medicare, Medicaid, and private insurers?

**Findings:**

In this cross-sectional study of 14 918 adults representing the US civilian, noninstitutionalized population with Medicare, Medicaid, or private insurance coverage, SDOH were significantly associated with US health care expenditures, with differential associations by insurer.

**Meaning:**

These findings suggest that SDOH may be used by health insurers and policymakers in their decision-making practices to identify and control health care expenditures, advancing health equity.

## Teaser

This cross-sectional study analyzes associations of various social determinants of health with health care expenditures among individuals with Medicare, Medicaid, or private insurance.

## Introduction

National health expenditures in the US have been growing rapidly, up 4.1% to $4.5 trillion in 2022, with 9.6% growth in Medicaid, 5.9% growth in Medicare, and 5.9% growth in private insurer spending.^1^ Over the next decade, average growth in health expenditures is projected to surpass average growth in gross domestic product.^1^ However, long-standing health inequities persist.^1^ The US federal government has committed to address social determinants of health (SDOH), the conditions in which individuals are born, live, learn, work, play, worship, and age, that are associated with a wide range of health, functioning, and quality of life risks and outcomes.^2^ The Healthy People 2030 initiative aims to create social, physical, and economic environments that promote the full potential for health and well-being for all, with structural SDOH categorized into 5 domains: (1) educational access and quality, (2) health care access and quality, (3) neighborhood and built environment, (4) economic stability, and (5) social and community context.^2^ At the individual level, health-related social needs (HRSN) are often direct manifestations of these community-level structural SDOH. Although previous studies have found associations of individual-level SDOH as HRSN with health outcomes,^2,3,4,5,6,7,8,9,10,11,12^ there is scarcity of evidence on SDOH and health care expenditures to make the economic argument for health insurers and public policymakers to target SDOH.

**Figure 1.**
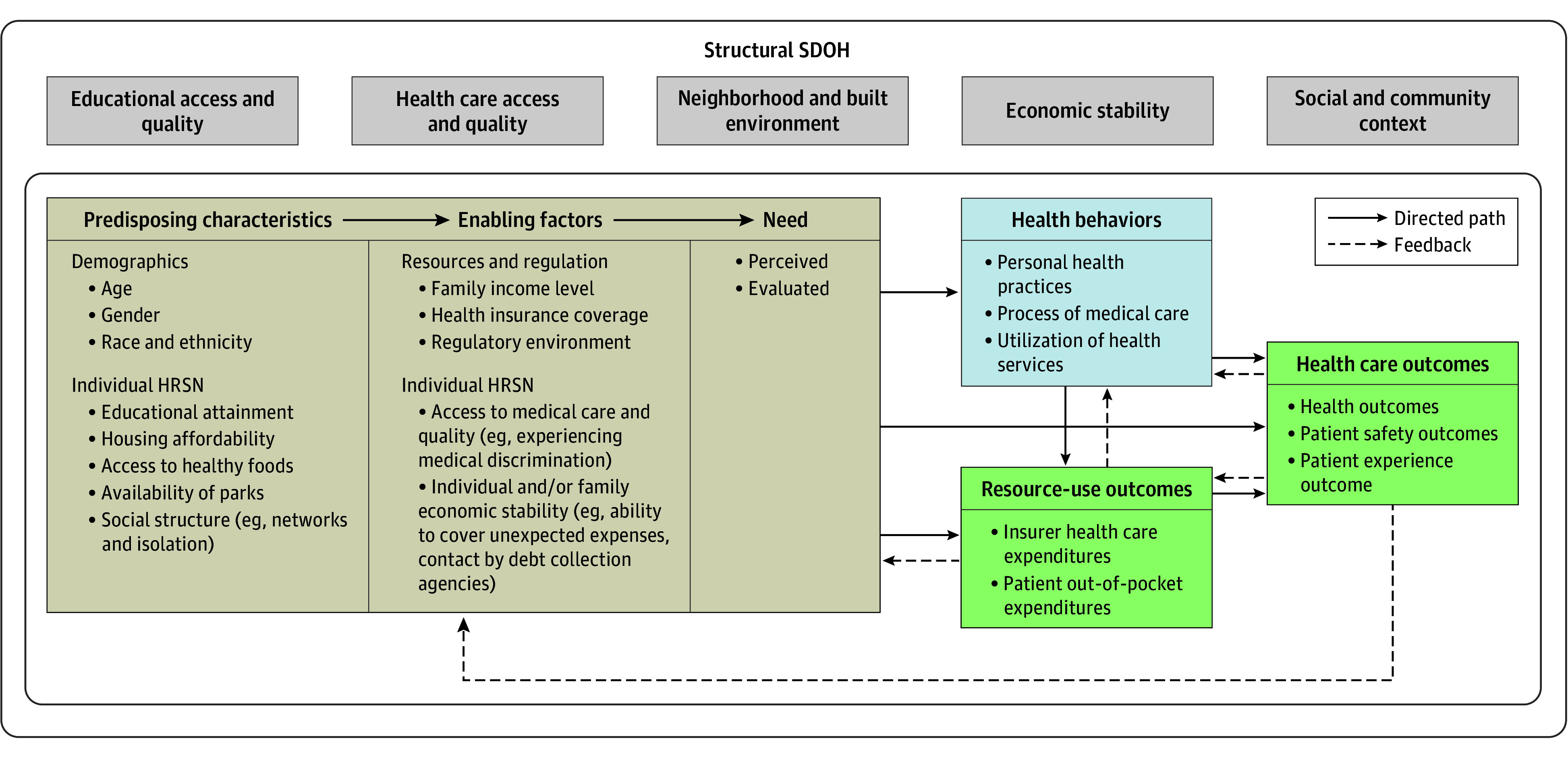
Conceptual Framework HRSN indicates health-related social needs; SDOH, social determinants of health.

We constructed a conceptual model, adapting the Anderson model for health utilization, which connects individual and contextual characteristics through predisposing, enabling, and need factors to health behaviors and subsequently health and resource-use outcomes (Figure 1).^13^ The outermost layer represents the broader structural SDOH domains at the community-level from Healthy People 2030, representing the conditions in which individuals live and thrive. Within this structural context, we superimposed a layer representing individual and contextual characteristics categorized into (1) factors that predispose individuals to utilize health services, (2) factors that may enable or hinder utilization of health services, (3) perceived and evaluated needs of how individuals and their clinicians view their health. There is likely to be dynamic interplay between predisposing and enabling factors at the individual level, which may work to influence health care needs and health behaviors (eg, preventive personal health practices and care-seeking), subsequently impacting health and resource-use outcomes. Feedback mechanisms likely exist between health and resource-use outcomes, behavior, and individual and contextual characteristics. For example, positive health outcomes may reinforce positive health behaviors like preventive care-seeking and potentially reduce future health care expenditures from early diagnosis and treatment of conditions. We specifically focused our study on examining the association of individual-level SDOH through HRSN with resource-use outcomes through insurer health care expenditures. Our study is well-suited to inform health insurers and policymakers on incorporating SDOH in their decision-making practices to identify and control health care expenditures, advancing health equity.

## Methods

### Study Design and Participants

This cross-sectional study was determined to be exempt from review and the requirement of informed consent by the Johns Hopkins University institutional review board because it used publicly available deidentified data. The reporting of the study follows the Strengthening the Reporting of Observational Studies in Epidemiology (STROBE) reporting guideline. We used data from the 2021 Medical Expenditure Panel Survey (MEPS), specifically the newly developed and released SDOH survey of the US noninstitutionalized civilian population. We included adults 18 years and older who had private, Medicare, or Medicaid coverage ever in 2021 and completed the SDOH survey (eFigure 1 in Supplement 1). We excluded individuals with other public coverage owing to low sample size (<1000 individuals) and uninsured individuals because our study aims to inform health insurers on whether to use SDOH in their decision-making. We performed a complete case analysis to handle missingness of variables. We assessed potential selection bias by comparing demographic characteristics of participants who completed the SDOH survey vs not and our sample before and after complete case analysis.

Our outcome was annual health care expenditures in US dollars by Medicare, Medicaid, and private insurers. We used the Healthy People 2030 SDOH domains to guide our selection of the following HRSN as individual-level SDOH exposures: educational attainment as the highest degree attained, focusing on high school completion (domain 1: education); access to medical care and medical discrimination as our measures for health care access and health care quality (domain 2), respectively; housing affordability, access to healthy foods, and availability of parks (domain 3: neighborhood and built environment); being contacted by collections in the past 12 months and ability to cover unexpected expenses (domain 4: economic stability); and social isolation and attending club or organization meetings per year (domain 5: social and community context).

### Statistical Analysis 

**Figure 2.**
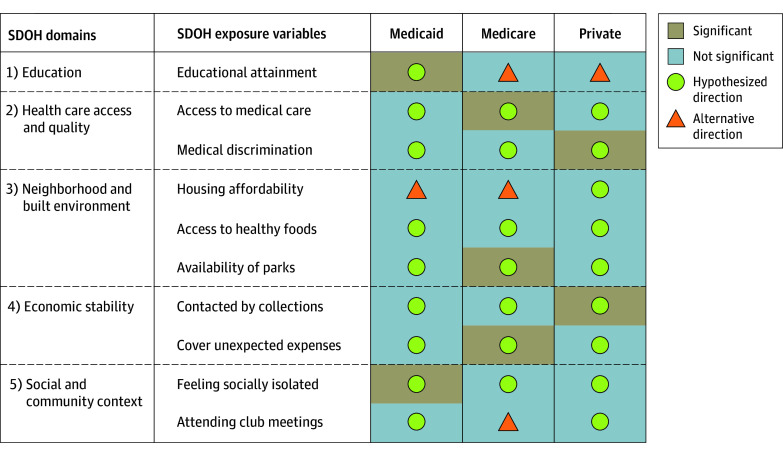
Association of Social Determinants of Health (SDOH) With Health Care Expenditures

Using our conceptual model, we identified self-reported age, gender, race and ethnicity, family income level, dual coverage of Medicare and Medicaid, and US census region (eg, regulatory environment) as confounders that may be associated with both exposure and outcome (eFigure 2 in Supplement 1). Family income level was categorized as percentage of federal poverty level, including poor (<100%), near poor (100%-124%), low (125%-199%), middle (200%-399%), and high (≥400%). Race and ethnicity categories included Hispanic, non-Hispanic Asian, non-Hispanic Black, non-Hispanic White, and non-Hispanic other (defined as American Indian, Alaskan Native, Pacific Islander, and/or multiracial); race and ethnicity were included as a social construct to account for disparities. Adjusting for these confounders enabled us to isolate the association of HRSN with insurer expenditures. We stratified our analysis by the type of insurer to be responsive to differences in demographic makeup of Medicare, Medicaid, and Private beneficiaries along with differential practices in plans and coverage elements. We developed 10 focused hypotheses about associations across the 5 SDOH domains considered to be the most informative for policy and practice. (eAppendix 1 in Supplement 1 and Figure 2). Participant characteristics were summarized as means with SDs for continuous variables, counts with frequency and percentages for dichotomous and categorical variables, and medians with IQRs for expenditures to be responsive to skewness.

We performed several diagnostic tests to guide model specification. Considering multiple exposures in our analysis, we performed the variance inflation factor (VIF) test for each independent variable to identify the degree of inflation in the variance of the regression coefficients attributable to the correlation among exposures. The Breusch-Pagan test for heteroskedasticity was used with null hypothesis of constant variance among residuals to check whether errors were uniform and identically distributed (homoscedastic). A 2-part econometric model was then fitted with the first part estimating whether there were any expenditures using a Probit model, and the second part estimating expenditures on nonzero observations using a generalized linear model (GLM). A Park test was performed to identify the family link function for the GLM. We performed multiple hypothesis testing covering our 10 focused hypotheses across 5 SDOH domains. Unconditional marginal effects were calculated accounting for both parts of the model. Our analysis was weighted to account for the complex sampling design of the MEPS survey. We used the Benjamin-Hochberg method to control the false discovery rate in our multiple hypothesis testing. Adjusted *P* values estimated from this process (2-sided) less than .05 were considered statistically significant. To assess whether associations varied significantly by insurer type, we fit a pooled 2-part model combining all 3 insurance groups with interaction terms between the insurer type and HRSN. An adjusted Wald test was used to test the joint significance of the interaction terms. Our analysis was performed in Stata version 17 (Stata Corp) with tabulation and visualization in R version 4.3.1 (R Project for Statistical Computing). Data analysis was conducted from October 2023 to April 2024.

## Results

### Demographics

Among the 14 918 insured adults in our analytic sample (mean [SD] age, 52.5 [17.9] years; 8471 female [56.8%]; 2477 Hispanic [16.6%]; 1947 non-Hispanic Black [13.1%]; 9235 non-Hispanic White [61.9%]), the majority had middle to high family income (10 524 participants [70.5%]) and were privately insured (10 227 participants [68.5%]) (Table 1). Among the 4691 (31.4%) who were publicly insured, 2644 (17.7%) had Medicaid, 2047 (13.7%) had Medicare, and 917 (6.1%) had dual coverage. Median (IQR) annual expenditures were highest for Medicare ($3643 [$1321-$10 519]) compared with Medicaid ($1648 [$389-$7126]) and private insurers ($1369 [$456-$4078]). Medicare beneficiaries were older (mean [SD] age, 72.0 [7.7] years) compared with Medicaid (mean [SD] age, 48.3 [17.8] years) and private beneficiaries (mean [SD] age, 49.6 [16.9] years). The majority of Medicare beneficiaries (1535 beneficiaries [75.0%]) and private beneficiaries (6653 beneficiaries [65.1%]) were non-Hispanic White, while 1408 Medicaid beneficiaries (53.2%) were Hispanic or non-Hispanic Black. The majority of Medicaid beneficiaries (1541 beneficiaries [58.2%]) were poor or near poor, while the majority of Medicare beneficiaries (1287 beneficiaries [62.9%]) were middle or high income and the majority of Private beneficiaries (5538 beneficiaries [54.2%]) were high income. US census region was distributed similarly across insurance groups. Distributions of HRSN are presented in eAppendix 2 in Supplement 1.

**Table 1.** Participant Characteristics by Insurance Beneficiary

Characteristic^a^	Participants, No. (%)			
	Medicare (n = 2047)	Medicaid (n = 2644)	Private (n = 10 227)	Overall (N = 14 918)
Insurer expenditures, median (IQR), $	3643 (1321−10 519)	1648 (389−7126)	1369 (456−4078)	1640 (509−5367)
Age, mean (SD), y	72.0 (7.7)	48.3 (17.8)	49.6 (16.9)	52.5 (17.9)
Sex				
Male	814 (39.8)	937 (35.4)	4696 (45.9)	6477 (43.2)
Female	1233 (60.2)	1707 (64.6)	5531 (54.1)	8471 (56.8)
Race and ethnicity				
Hispanic	196 (9.6)	779 (29.5)	1502 (14.7)	2477 (16.6)
Non-Hispanic Asian	58 (2.8)	84 (3.2)	683 (6.7)	825 (5.5)
Non-Hispanic Black	215 (10.5)	629 (23.8)	1103 (10.8)	1947 (13.1)
Non-Hispanic White	1535 (75.0)	1047 (39.6)	6653 (65.1)	9235 (61.9)
Non-Hispanic other^b^	43 (2.1)	105 (3.9)	286 (2.8)	434 (2.9)
Family income level^c^				
Poor (<100%)	266 (13.0)	1222 (46.2)	481 (4.7)	1969 (13.2)
Near poor (100%-124%)	131 (6.4)	319 (12.1)	216 (2.1)	666 (4.5)
Low (125%-199%)	363 (17.7)	479 (18.1)	917 (8.9)	1759 (11.8)
Middle (200%-399%)	612 (29.9)	475 (17.9)	3075 (30.1)	4162 (27.9)
High (≥400%)	675 (32.9)	149 (5.6)	5538 (54.2)	6362 (42.7)
US census region				
Northeast	334 (16.3)	453 (17.1)	1651 (16.1)	2438 (16.3)
Midwest	431 (21.1)	485 (18.3)	2259 (22.1)	3175 (21.3)
South	739 (36,1)	928 (35.1)	3609 (35.3)	5276 (35.4)
West	543 (26.5)	778 (29.4)	2708 (26.5)	4029 (27.0)
Educational attainment				
Less than high school	205 (10.0)	829 (31.4)	699 (6.9)	1733 (11.6)
High school diploma or GED	647 (31.6)	1008 (38.1)	2351 (23.0)	4006 (26.9)
Some college	487 (23.8)	534 (20.2)	2405 (23.5)	3426 (23.0)
Bachelor’s degree and higher	708 (34.6)	273 (10.3)	4772 (46.7)	5753 (28.6)
Experienced medical discrimination	112 (5.5)	341 (12.9)	808 (7.9)	1261 (8.5)
Access to medical care				
Excellent	838 (40.9)	671 (25.4)	3821 (37.4)	5330 (35.7)
Very good	719 (35.1)	817 (30.9)	3693 (36.1)	5229 (35.1)
Good	348 (17.0)	767 (29.0)	1931 (18.9)	3046 (20.4)
Fair	104 (5.1)	309 (11.7)	589 (5.8)	1002 (6.7)
Poor	38 (1.9)	80 (3.0)	193 (1.9)	311 (2.1)
Housing affordability				
Excellent	207 (10.1)	337 (12.8)	801 (7.8)	1345 (9.0)
Very good	424 (20.7)	408 (15.4)	1903 (18.6)	2735 (18.3)
Good	681 (33.3)	775 (29.3)	3481 (34.0)	4937 (33.1)
Fair	477 (23.)	656 (24.8)	2643 (25.8)	3776 (25.3)
Poor	258 (12.6%)	468 (17.7)	1399 (13.7)	2125 (14.2)
Access to healthy foods				
Excellent	736 (36.0)	614 (23.2)	3451 (33.7)	4801 (32.2)
Very good	723 (35.3)	763 (28.9)	3483 (34.1)	4969 (33.3)
Good	395 (19.3)	748 (28.3)	2127 (20.8)	3270 (21.9)
Fair	135 (6.6)	358 (13.5)	823 (8.1)	1316 (8.8)
Poor	58 (2.8)	161 (6.1)	343 (3.4)	562 (3.8)
Availability of parks				
Excellent	639 (31.2)	578 (21.9)	3509 (34.3)	4726 (31.7)
Very good	664 (32.4)	711 (26.9)	3392 (33.2)	4767 (32.0)
Good	496 (24.2)	723 (27.3)	2111 (20.6)	3330 (22.3)
Fair	162 (7.9)	428 (16.2)	888 (8.7)	1478 (9.9)
Poor	86 (4.2)	204 (7.7)	327 (3.2)	617 (4.1)
Contacted by collection agencies in past 12 mo	232 (11.3)	730 (27.)	1393 (13.6)	2355 (15.8)
Ability to cover unexpected expenses				
Not confident	390 (19.1)	1440 (54.5)	1644 (16.1)	3474 (23.3)
Somewhat confident	457 (22.3)	796 (30.1)	2389 (23.4)	3642 (24.4)
Very confident	1200 (58.6)	408 (15.4)	6194 (60.6)	7802 (52.3)
Attending club or organization meetings, No. of times/y				
Never	1232 (60.2)	2077 (78.6)	6028 (58.9)	9337 (62.6)
1-9	398 (19.)	383 (14.5)	2409 (23.6)	3190 (21.4)
10-15	170 (8.3)	64 (2.4)	683 (6.7)	917 (6.2)
≥16	247 (12.1)	120 (4.5)	1107 (10.8)	1474 (9.8)
Feeling isolated				
Never	821 (40.1)	1190 (38.5)	3796 (37.1)	5635 (37.8)
Rarely	574 (28.0)	645 (20.9)	3113 (30.4)	4255 (28.5)
Sometimes	481 (23.5)	809 (26.2)	2501 (24.5)	3676 (24.6)
Often	171 (8.4)	419 (13.6)	817 (8.0)	1352 (9.1)

Abbreviation: GED, general educational development.

^a^ Race and ethnicity as well as all other variables were self-reported by participants in the 2021 Medical Expenditure Panel Survey.

^b^ The non-Hispanic other race and ethnicity category represents non-Hispanic individuals including American Indian, Alaskan Native, Pacific Islander, and/or multiracial individuals.

^c^ Family income level is categorized based on percentage of federal poverty level.

### Diagnostics

We found minimal risk for selection bias from SDOH survey completion and complete case analysis (eTable 1 and eTable 2 in Supplement 1). Our pooled analysis (eTable 3 in Supplement 1) found significant differences in the associations of HRSN with insurer expenditures across the 3 insurance beneficiary groups, further warranting our stratification of the analysis by health insurer. The VIF test (eTable 4 in Supplement 1) found minimal multicollinearity not expected to impact the reliability of our estimated coefficients based on a mean (SD) VIF of 1.31 (0.36) with each individual exposure less than 2.1 against the threshold for concern of VIF (≥10). The Breusch-Pagan test for heteroskedasticity (eTable 5 in Supplement 1) found significant evidence of nonconstant variance in the residuals in the preliminary linear model. The Park test (eTable 6 in Supplement 1) found a slope coefficient of 1.99 (95% CI, 1.83-2.14), close to 2 indicating variance proportional to the square of the mean, which suggests that a γ distribution with a log-link is well suited for our GLM. This specification inherently does not assume constant variance across all observations, handling the heteroskedasticity we observed.

### Medicaid Expenditures

The unconditional marginal effects from our 2-part model with adjusted *P* values using Benjamin-Hochberg method for multiple hypothesis testing are presented in Table 2 for expenditures by Medicare, Medicaid, and Private insurers with visualization in Figure 2. Medicaid beneficiaries who attained a high school diploma or general educational development (GED) certificate had on average (mean difference) $2245.39 lower annual Medicaid expenditures (95% CI, −$3700.97 to −$789.80) compared with Medicaid beneficiaries with less than a high school education (adjusted *P* = .02). Medicaid expenditures were on average $2706.94 (95% CI, $1339.06 to $3074.82) higher annually for beneficiaries who often felt isolated compared with beneficiaries who never felt isolated (adjusted *P* < .001). Associations for other exposures (eg, access to medical care, medical discrimination, access to healthy foods, availability of parks, contacted by collections, ability to cover unexpected expenses, and attending club or organization meetings) appeared to exhibit the hypothesized direction of effect, but were not considered to be statistically significant.

**Table 2.** Two-Part Modeling Results by Health Insurer^a^

Exposure by by SDOH domain	Medicaid expenditures^b^			Medicare expenditures^c^			Private insurer expenditures^d^		
	Difference estimate, mean (95% CI), $	*P* value		Difference estimate, mean (95% CI), $	*P* value		Difference estimate, mean (95% CI), $	*P* value	
		Unadj^e^	Adj^f^		Unadj^e^	Adj^f^		Unadj^e^	Adj^f^
1. Educational attainment									
High school diploma or GED vs less than high school	−2245.39 (−3700.97 to −789.80)^g^	.003	.02	1586.04 (−1482.37 to 4654.46)	.31	.52	751.67 (−937.20 to 2440.55)	.38	.50
2. Health care access and quality									
Access to medical care (poor vs excellent)	−2966.49 (−5425.58 to −507.39)	.02	.06	−7653.47 (−11 733.21 to −3573.72)^g^	<.001	<.001	−2020.93 (−4144.28 to 102.42)	.06	.16
Medical discrimination (yes vs no)	197.79 (−945.18 to 1340.76)	.73	.73	468.30 (−3020.22 to 3956.81)	.79	.79	2599.93 (863.71 to 4336.15)^g^	.003	.02
3. Neighborhood and built environment									
Housing affordability (poor vs excellent)	−2680.19 (−5069.99 to −290.37)	.03	.07	−1222.70 (−5712.18 to 3266.77)	.59	.74	357.55 (−1095.08 to 1810.19)	.63	.70
Access to healthy foods (poor vs excellent)	3693.73 (298.12 to 7089.34)	.03	.07	2696.78 (−4265.48 to 9659.04)	.45	.64	1963.41 (−2146.83 to 6073.65)	.35	.50
Availability of parks (good vs excellent)	635.00 (−1042.74 to 2312.75)	.46	.57	5959.27 (1679.99 to 10 238.55)^g^	.007	.03	442.14 (−589.38 to 1473.65)	.40	.50
4. Economic stability									
Contacted by collections in past 12 mo (yes vs no)	699.24 (−285.13 to 1683.60)	.16	.27	2922.98 (−303.31 to 6149.27)	.08	.15	2033.34 (896.82 to 3169.86)^g^	.001	.01
Ability to cover unexpected expenses (confident vs not)	−529.94 (−1673.56 to 613.68)	.36	.52	−3743.98 (−6500.68 to −987.28)^g^	.008	.03	−227.83 (−1380.42 to 924.76)	.70	.70
5. Social and community context									
Feeling socially isolated (often vs never)	2706.94 (1339.06 to 4074.82)^g^	<.001	<.001	9837.95 (81.39 to 19 594.51)	.048	.12	1784.46 (29.01 to 3539.90)	.046	.15
Attending club or organization meetings (10-15 times/y vs never)	−476.11 (−2821.81 to 1869.59)	.69	.73	714.39 (−2750.80 to 4179.58)	.69	.76	−553.34 (−1421.58 to 314.91)	.21	.42

Abbreviations: Adj, adjusted; GED, general educational development; SDOH, social determinants of health; Unadj, unadjusted.

^a^ The first part estimates whether there were any expenditures using a Probit model: Pr(Expenditures >0 / *X*) = Φ(β_0_ + β_x_ × [*X*] + β_z_ × [*Z*]), where *X* is the vector of exposures and Z is the vector of confounders. The second part estimates expenditures on nonzero observations using a generalized linear model with log-link: E(Expenditures / Expenditures >0, *X*) = exp(α_0_ + α_x_ × [*X*] + α_z_ × [*Z*]).

^b^ Model 1: Total Medicaid expenditures (outcome) with SDOH variables listed above as primary exposures, adjusted for age, gender, race and ethnicity, family poverty level, US census region, having dual coverage of Medicare and Medicaid.

^c^ Model 2: Total Medicare expenditures (outcome) with SDOH variables listed above as primary exposures, adjusted for age, gender, race and ethnicity, family poverty level, US census region, having dual coverage of Medicare and Medicaid.

^d^ Model 3: Total Private insurer expenditures (outcome) with SDOH variables listed above as primary exposures, adjusted for age, gender, race and ethnicity, family poverty level, US census region.

^e^ Unadjusted *P* values before multiple hypothesis testing correction.

^f^ Adjusted *P* values after performing Benjamin-Hochberg method for multiple hypothesis testing.

^g^ Adjusted *P* < .05 considered statistically significant.

### Medicare Expenditures

Medicare beneficiaries with poor access to medical care had on average (mean difference) $7653.47 lower annual Medicare expenditures (95% CI, −$11 733.21 to −$3573.72) compared with beneficiaries with excellent access to medical care (adjusted *P* < .001). Medicare beneficiaries living in neighborhoods with lower availability of parks had on average $5959.27 (95% CI, $1679.99 to $10 238.55) higher annual Medicare expenditures than those with higher availability (adjusted *P* = .03). Medicare beneficiaries who were confident in their ability to cover unexpected expenses had on average $3743.98 lower annual Medicare expenditures (95% CI, −$6500.68 to −$987.28) than beneficiaries who were not confident (adjusted *P* = .03). Associations for other exposures (eg, medical discrimination, access to healthy foods, contacted by collections, and feeling socially isolated) appeared to exhibit the hypothesized direction of effect, but were not considered to be statistically significant.

### Private Expenditures

Private insurance beneficiaries who experienced medical discrimination had on average (mean difference) $2599.93 (95% CI, $863.71- $4336.15) higher annual private insurer expenditures compared with beneficiaries who did not experience medical discrimination (adjusted *P* = .02). Private expenditures were on average $2033.34 (95% CI, $896.82-$3169.86) higher annually for beneficiaries who were contacted by debt collections compared with those who were not contacted in the past 12 months (adjusted *P* = .01). Associations for the other exposures (eg, access to medical care, housing affordability, access to healthy foods, availability of parks, ability to cover unexpected expenses, feeling socially isolated, and number of times attending club or organization meetings per year) appeared to exhibit the hypothesized direction of effect, but were not considered to be statistically significant.

## Discussion

### Summary of Findings

This cross-sectional study is the first, to our knowledge, to empirically quantify the association of SDOH with US health care expenditures by insurer to inform policy and practice. We found SDOH as individual-level HRSN to be significantly associated with expenditures by Medicare, Medicaid, and private insurers. Educational attainment and social isolation were associated with Medicaid expenditures. Attaining a high school diploma or GED was associated with lower Medicaid expenditures compared with no degree. Medicaid beneficiaries who often experienced social isolation had on average higher Medicaid expenditures compared with beneficiaries who never felt isolated. Health care access, built environment, and economic stability were associated with Medicare expenditures. Reduced availability of parks in the neighborhood was associated with higher Medicare expenditures. The ability to cover unexpected expenses was associated with lower Medicare expenditures. Medical discrimination and economic stability were associated with higher private expenditures. Medical discrimination as an indicator of trust and proxy for health care quality was associated with greater private insurance expenditures. Private beneficiaries contacted by collections had higher private expenditures.

All 7 statistically significant associations we observed were in the hypothesized direction. Other associations alternative to our hypotheses were not statistically significant. The differential associations we observed likely reflect the unique characteristics and needs for each insurance group. For example, Medicaid beneficiaries are often younger and poorer and may experience more acute social challenges like educational attainment. In contrast, Medicare beneficiaries, often being older, may benefit more from built environment (eg, access to parks) for physical activity for chronic disease management and social interaction. Private beneficiaries’ higher out-of-pocket costs may contribute to higher quality expectations, making them especially sensitive to medical discrimination. Economic stability was significant in both Medicare and private beneficiaries, highlighting different financial pressures faced by these groups. Medicare beneficiaries may be focused on fixed incomes after retirement while private beneficiaries may be focused on dealing with employment instability and debt. We recognize the resource constraints insurers face that may necessitate a more focused approach in the shorter term. Insurers may initially prioritize the SDOH domains found to be significant in our analysis and then expand efforts to the broader range of structural SDOH fueling health inequities.

### Proposed Approaches

There are a wide range of options health insurers have in using SDOH to guide their decision-making processes to manage expenditures and promote health equity. SDOH may be addressed at the individual level through HRSN (eg, addressing an individual person’s access to healthy foods) and/or at the community level through structural SDOH (eg, addressing community level access to healthy foods). While addressing HRSN offers a short-term approach, addressing structural SDOH offers a longer-term and potentially more sustainable solution to tackle the root cause of health inequities.

At the individual level, insurers may consider the use of value payment models to incentivize clinicians to screen for HRSN, promote team-based care that incorporate behavioral and social needs, and rewards clinicians who connect individuals with HRSN with appropriate community-based services. They may also provide tools and training for clinicians within their networks to improve integration of HRSN screening into their standard health care practice. For example, insurers may opt to deliver cost-effective virtual training to clinicians on preventing medical discrimination and biases in their clinical interactions and offer incentives for training completion and integration into care. Quality assessments through virtual surveys of beneficiaries may be used to further reinforce and reward these positive clinician behaviors. Insurers may also use HRSN as a tool to identify potential high spenders among their beneficiary pool to more effectively target interventions (eg, incentives) that encourage preventive practices to reduce more expensive longer-term expenditures from delayed diagnosis complications.

At the community level, insurers may consider participating in a multisectoral consortia consisting of key stakeholders from public and private sectors to collaboratively address structural SDOH, the root cause of health inequities. Insurers are encouraged to advocate for the integration of health considerations into policymaking across various sectors through the federal and state Health in All Policies initiative. Partnering with advocacy groups to encourage and support policy-oriented solutions from public policymakers may enhance efforts addressing structural SDOH, advancing health equity.

### Limitations

This study has limitations. We are unable to establish causality owing to the cross-sectional nature of our study. However, we identified beneficiaries at risk for increased health care expenditures based on HRSN. Future studies using longitudinal designs may be beneficial in establishing temporal associations. We did not have access to a measure for educational quality using the survey data and used only medical discrimination to capture health care quality. Improvements in standardized measurement of SDOH variables may promote consistency across studies to better articulate these complex associations. The self-reported data may introduce sources of information bias, especially recall bias during the interviews in which participants may not accurately recall past behaviors, events, or attributes. Social desirability bias may arise from participants providing responses they believe to be socially acceptable.

SDOH may be associated with the structure of health benefit plans (eg, traditional Medicare with its deductible and copay system, Medicare Advantage bundled pricing, and higher deductible private plans). Insurer-driven costs are predominantly driven by utilization (quantity) rather than pricing (per-unit cost) because insurers typically negotiate service prices. MEPS does not provide detailed structure of benefit plans, limiting our ability to decompose the observed association attributable to utilization vs benefit structure. In addition, health expenditures are likely correlated with market power of health care systems and geography. We were able to adjust for geography broadly with US census region. However, lack of availability of variables such as health care system market power and benefit design in MEPS may contribute to potential unmeasured confounding. Residual confounding may remain even after our adjustment for measured confounders. Our findings may be limited to US civilian, noninstitutionalized population with Medicare, Medicaid, or private coverage and may not be generalizable to populations outside of this context, including individuals who are institutionalized, uninsured, and with other sources of public insurance coverage.

## Conclusions

This cross-sectional study found individual-level SDOH to be significantly associated with US health care expenditures, potentially incentivizing health insurers to utilize SDOH in their decision-making practices to identify and control expenditures. Health insurers may use HRSN to identify beneficiaries at greater risk for high expenditures to target interventions by prioritizing SDOH domains found to be significant in our analysis. Addressing structural SDOH may require insurers to engage with multisectoral stakeholders with shared funding mechanisms and for public policymakers to adopt a health-in-all policies approach. While addressing HRSN may be more feasible in the short term, targeting structural SDOH through multisectoral partnerships may address the root cause to achieve a more equitable and sustainable health care system.

## Notes

Supplement 1. **eFigure 1.** Analytic Sample Inclusion Criteria **eFigure 2.** Analytic Diagram **eAppendix 1.** Focused Hypotheses **eAppendix 2.** Participant Distribution of HRSN by SDOH Domain **eTable 1.** Participants Who Did Not Complete SDOH Survey **eTable 2.** Sample Before Complete Case Analysis **eTable 3.** Pooled Model Joint Significance Testing **eTable 4.** Variance Inflation Test (VIF) Results **eTable 5.** Breusch-Pagan Test for Heteroskedasticity Results **eTable 6.** Park Test Results

Supplement 2.
Data Sharing Statement
