# Supplementary material for: Social Determinants of Health and US Health Care Expenditures by Insurer
Source: JAMA Netw Open. 2024 Oct 23;7(10):e2440467. doi: 10.1001/jamanetworkopen.2024.40467 (PMC11581502; doi:10.1001/jamanetworkopen.2024.40467)
Supplement: Supplement 1. — eFigure 1. Analytic Sample Inclusion Criteria eFigure 2. Analytic Diagram eAppendix 1. Focused Hypotheses eAppendix 2. Participant Distribution of HRSN by SDOH Domain eTable 1. Participants Who Did Not Complete SDOH Survey eTable 2. Sample Before Complete Case Analysis eTable 3. Pooled Model Joint Significance Testing eTable 4. Variance Inflation Test (VIF) Results eTable 5. Breusch-Pagan Test for Heteroskedasticity Results eTable 6. Park Test Results [file jamanetwopen-e2440467-s001.pdf]

Supplemental Online Content

Mohan G, Gaskin DJ. Social determinants of health and US health care expenditures by insurer. *JAMA Netw. Open*. 2024;7(10):e2440467.
doi:10.1001/jamanetworkopen.2024.40467

eFigure 1. Analytic Sample Inclusion Criteria
eFigure 2. Analytic Diagram
eAppendix 1. Focused Hypotheses
eAppendix 2. Participant Distribution of HRSN by SDOH Domain
eTable 1. Participants Who Did Not Complete SDOH Survey
eTable 2. Sample Before Complete Case Analysis
eTable 3. Pooled Model Joint Significance Testing
eTable 4. Variance Inflation Test (VIF) Results
eTable 5. Breusch-Pagan Test for Heteroskedasticity Results
eTable 6. Park Test Results

This supplemental material has been provided by the authors to give readers additional information about their work.

© 2024 Mohan G et al. *JAMA Network Open.*

eFigure 1. Analytic Sample Inclusion Criteria

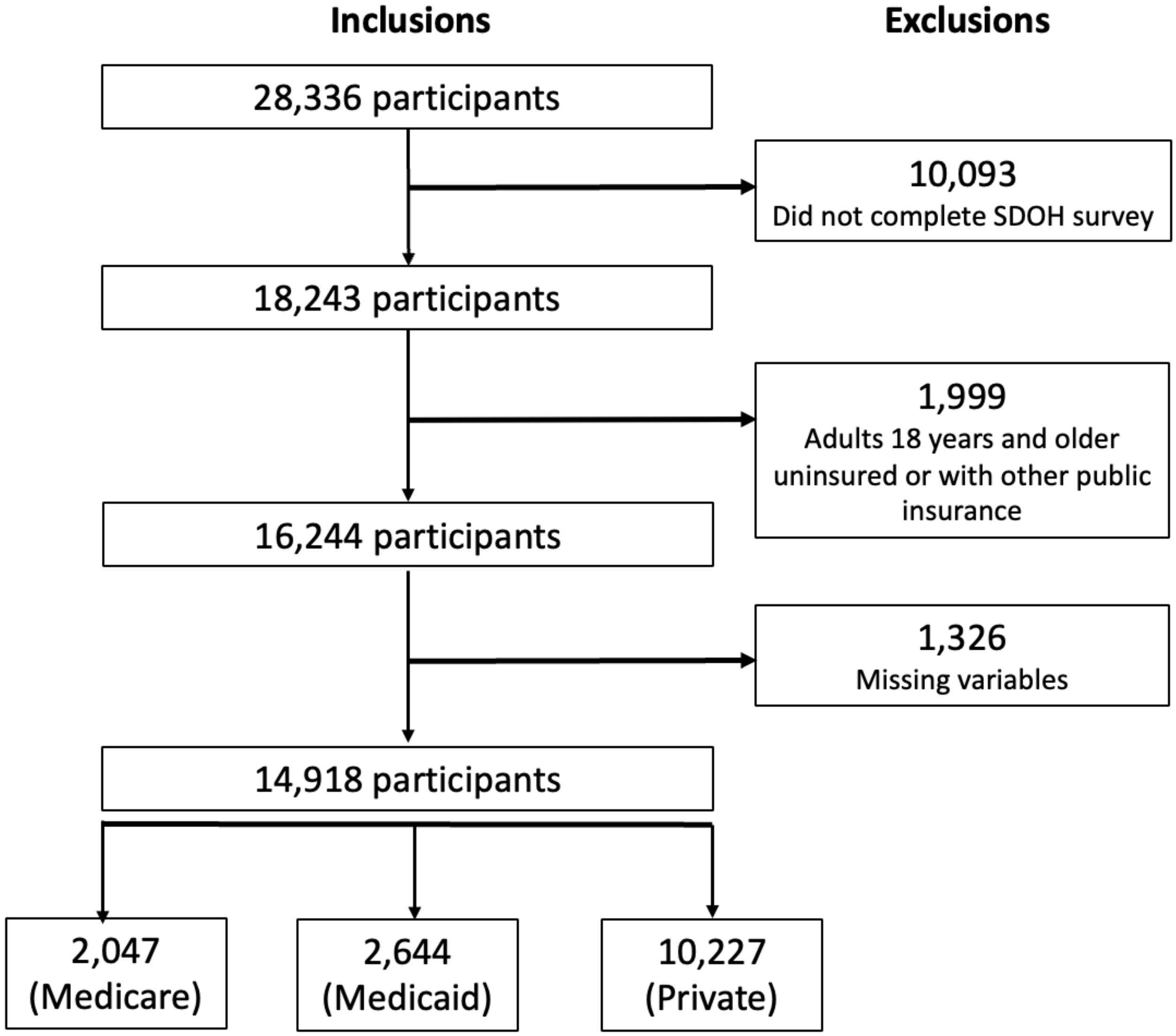

From 28,336 individuals in the 2021 MEPS Survey, we included 14,918 individuals in our analytic sample representing adults 18 years and older who completed the SDOH survey, had Medicare, Medicaid, or Private insurance coverage in 2021, and did not have any missing exposure, covariate, or outcome variables. Of the 14,918 individuals, 10,227 had Private (68%), 2,047 had Medicare (14%), and 2,644 had Medicaid (18%).

© 2024 Mohan G et al. *JAMA Network Open.*

eFigure 2. Analytic Diagram

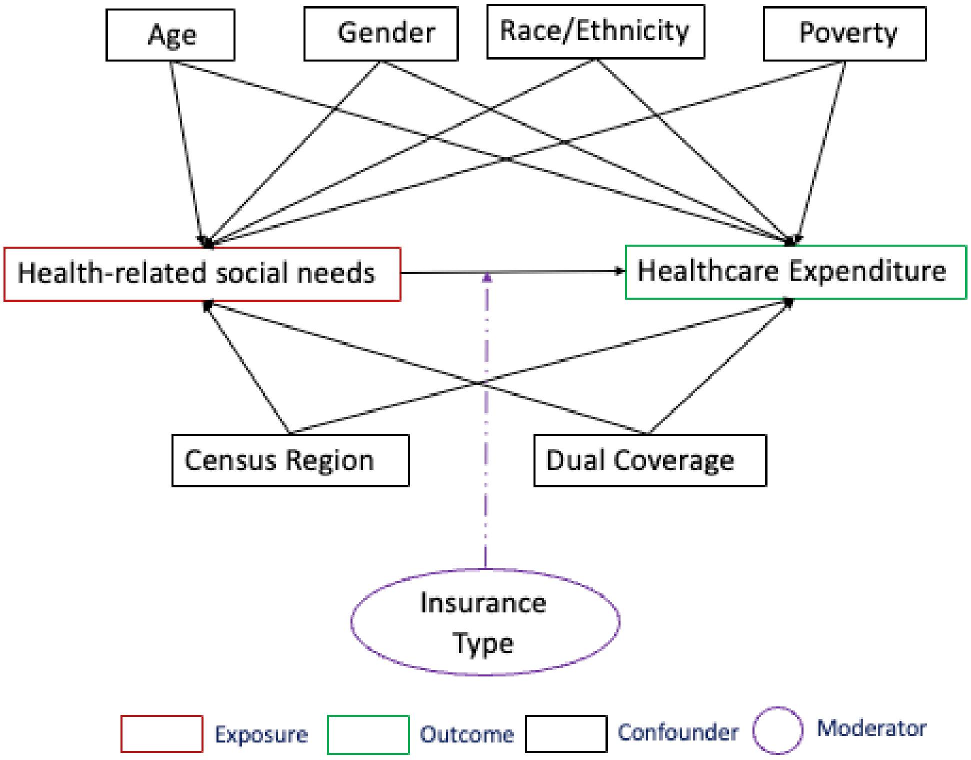

We used the existing domains for SDOH structured by Healthy People 2030 to guide our selection of HRSN exposure variables. For Domain 1 on Educational access and quality, we used educational attainment as the highest degree attained by the individual. We were unable to find an appropriate measure for educational quality, so we did not focus on the second element of Domain 1 in our analysis. For Domain 2 on Healthcare access and quality, we used access to medical care as our measure for healthcare access and medical discrimination as our measure for healthcare quality. For Domain 3 on Neighborhood and Built Environment, we used the following existing categorical measures (excellent to poor) as exposures: housing affordability, access to healthy foods, and availability of parks in the neighborhood. For Domain 4 on

© 2024 Mohan G et al. *JAMA Network Open.*

Economic Stability, we used the following as exposures: being contact by collections in the past 12 months and ability to cover unexpected expenses. For Domain 5 on Social and Community Context, we used the following as exposures: feeling isolated and attending club or organization meetings per year. We did not create composite scores for each domain. Instead, we used each of these exposure variables individually to gain insight on which would be beneficial to target for respective beneficiaries.

Each of the confounders we selected is likely to be associated with both the exposure (individual level SDOH as HRSN) and healthcare expenditures. Age is associated with HRSN, healthcare needs, utilization, and expenditures. For example, younger individuals are more likely to face challenges with educational attainment (HRSN), which may impact their income and utilization of preventive care services leading to greater expenditures from delayed diagnosis in the future. For gender, women may experience different healthcare access (HRSN) challenges compared to men, which may lead to differing healthcare expenditures. For race/ethnicity, minority populations may face more medical discrimination (HRSN), leading to lower utilization of preventive care services and leading to delayed care that may have greater expenditures. In our analysis, we used race/ethnicity as a social construct and acknowledge that it is highly associated with SDOH. Controlling for racism through race/ethnicity enables us to isolate the direct impact of HRSN on healthcare expenditures. For family poverty level, individuals from lower income families are likely to have greater challenges with housing affordability (HRSN) and may have to live in housing with potential health hazards. For example, housing with potential lead and mold exposures are known to be associated with exacerbating health conditions, leading to greater healthcare expenditures. For U.S. census region, regulation and policies vary by region, which may impact access to care and neighborhood attributes like access to healthy foods and parks.

© 2024 Mohan G et al. *JAMA Network Open.*

Individuals living in food deserts may not have the opportunity to make healthy dietary decisions, leading to the development and progression of chronic diseases that have greater expenditures. For dual Medicare-Medicaid coverage, these individuals may experience challenges navigating access to medical care (HRSN) owing to potentially fragmented systems of care, which might contribute to utilization and potentially greater expenditures from delayed diagnosis of conditions.

© 2024 Mohan G et al. *JAMA Network Open.*

eAppendix 1. Focused Hypotheses

Using out measured exposure variables, we created the following 10 focused hypotheses for relationships that may be most information to health insurers and public policymakers:

1. Hypothesis 1 (Domain 1): Individuals who attained a high school diploma or GED are hypothesized to have lower healthcare expenditures compared to individuals with less than high school attainment.
2. Hypothesis 2 (Domain 2): Individuals who have poor access to medical care are hypothesized to have lower healthcare expenditures compared to individuals with excellent access to medical care.
3. Hypothesis 3 (Domain 2): Individuals who experienced medical discrimination are hypothesized to have higher healthcare expenditures compared to individuals who did not experience medical discrimination.
4. Hypothesis 4 (Domain 3): Individuals who live in neighborhoods with poor housing affordability are hypothesized to have higher healthcare expenditures compared to individuals who live in neighborhoods with excellent housing affordability.
5. Hypothesis 5 (Domain 3): Individuals who live in neighborhoods with poor access to healthy foods are hypothesized to have higher healthcare expenditures compared to individuals who live in neighborhoods with excellent access to healthy foods.
6. Hypothesis 6 (Domain 3): Individuals who live in neighborhoods with good availability of parks are hypothesized to have higher healthcare expenditures compared to individuals who live in neighborhoods with excellent availability of parks.

© 2024 Mohan G et al. *JAMA Network Open.*

7. Hypothesis 7 (Domain 4) : Individuals who were contacted by collections in the past 12 months are hypothesized to have higher healthcare expenditures compared to individuals who were not contacted by collection agencies.
8. Hypothesis 8 (Domain 4): Individuals who were confident in their ability to cover unexpected expenses are hypothesized to have lower healthcare expenditures compared to individuals who were not confident in their ability to cover unexpected expenses.
9. Hypothesis 9 (Domain 5): Individuals who often felt socially isolated versus never are hypothesized to have higher healthcare expenditures.
10. Hypothesis 10 (Domain 5): Individuals who attended 10-15 club or organizational meetings per year are hypothesized to have lower healthcare expenditures compared to individuals who never attended these meetings.

© 2024 Mohan G et al. *JAMA Network Open.*

eAppendix 2. Participant Distribution of HRSN Variables by SDOH Domain

<u>SDOH Domain 1 (Education):</u> 58% of Medicare beneficiaries and 70% of Private beneficiaries had at least some college or higher with 70% of Medicaid beneficiaries having high school diploma or less.

<u>SDOH Domain 2 (Healthcare access and quality):</u> Access to medical care was similar across insurance groups with slightly lower access among Medicaid beneficiaries. A higher proportion of Medicaid beneficiaries experienced medical discrimination (13%) compared with Medicare (6%) and Private beneficiaries (8%).

<u>SDOH Domain 3 (Neighborhood and Built Environment):</u> A higher proportion of Medicaid beneficiaries had poor housing affordability (18%) compared ith Medicare (13%) and Private beneficiaries (14%). A higher proportion of Medicaid beneficiaries had poor access to healthy foods (6%) compared ith Medicare (3%) and Private beneficiaries (3%). A higher proportion of Medicaid beneficiaries had poor availability of parks in their neighborhoods (8%) compared ith Medicare (4%) and Private beneficiaries (3%).

SDOH Domain 4 (Economic Stability): A higher proportion of Medicaid beneficiaries were contacted by debt collection agencies in the past 12 months (28%) compared with Medicare (11%) and Private beneficiaries (14%). Similarly, majority of Medicaid beneficiaries were not

© 2024 Mohan G et al. *JAMA Network Open.*

confident in their ability to cover unexpected expenses (55%) compared with Medicare (19%) and Private beneficiaries (16%).

SDOH Domain 5 (Social and Community Context): Majority of all three insurance groups never attended club or organization meetings in the past year. A higher proportion of Medicaid beneficiaries (14%) often felt socially isolated compared with Medicare (8%) and Private beneficiaries (8%).

© 2024 Mohan G et al. *JAMA Network Open.*

eTable 1. Participants Who Did Not Complete SDOH Survey (N =3,390)

	Medicare (N=390)	Medicaid (N=699)	Private (N=2301)	Overall (N=3,390)
**Age**				
Mean (SD), y	73.6 (8.3)	46.4 (18.9)	44.3 (17.2)	47.4 (18.9)
**Sex**				
Male	166 (42.6%)	326 (46.6%)	1267 (55.1%)	2129 (53.4%)
Female	224 (57.4%)	373 (53.4%)	1034 (44.9%)	1856 (46.6%)
**Race and ethnicity**				
Hispanic	56 (14.4%)	233 (33.3%)	458 (19.9%)	1041 (26.1%)
Non-Hispanic Asian	16 (4.1%)	29 (4.2%)	143 (6.2%)	210 (5.3%)
Non-Hispanic Black	72 (18.5%)	179 (25.6%)	341 (14.8%)	695 (17.4%)
Non-Hispanic White	239 (61.3%)	228 (32.6%)	1275 (55.4%)	1906 (47.8%)
Non-Hispanic Other	7 (1.8%)	30 (4.3%)	84 (3.7%)	133 (3.3%)
**Family income level**				
Poor (<100%)	79 (20.3%)	291 (41.6%)	144 (6.3%)	662 (16.6%)
Near Poor (100-124%)	36 (9.2%)	80 (11.4%)	39 (1.7%)	194(4.9%)
Low (125-199%)	74 (18.9%)	133 (19.0%)	228 (9.9%)	538 (13.5%)
Middle (200-399%)	109 (27.9%)	137 (19.6%)	721 (31.3%)	1170 (29.4%)
High (≥400%)	92 (23.6%)	58 (8.3%)	1169(50.8%)	1421 (35.7%)
**U.S. Census Region**				
Northeast	67 (19.5%)	186 (27.5%)	388 (17.2%)	700 (18.2%)
Midwest	61 (17.8%)	88 (13.0%)	385 (17.1%)	599 (15.6%)
South	140 (40.8%)	206 (30.4%)	839 (37.2%)	1491 (38.7%)
West	75 (21.9%)	197 (29.1%)	643 (28.5%)	1063 (27.6%)

The Agency for Health Care Quality and Research developed the SDOH survey using validated federal surveys and instruments in consultation with multisectoral subject matter experts. The SDOH survey was first released in 2021, the most recent iteration of the MEPS data. Of the 28,336 individuals that completed the MEPS survey, 18,243 individuals (64.4%) completed the SDOH survey. Among the 10,093 individuals that did not take the SDOH survey,

© 2024 Mohan G et al. *JAMA Network Open.*

3,390 individuals were adults 18 years with Medicare, Medicaid, or Private insurance coverage in 2021. We examined the demographic characteristics of the insured adults who did not complete the SDOH survey (N = 3,390) with our analytic sample of insured adults who completed the SDOH survey (N = 14,918) for any differences.

The demographic characteristics of the insured adults who did not complete the SDOH survey were quite similar to those who did in our analytic sample, indicating minimal risk for selection bias. The mean age was similar in both samples across the three insurance groups: Medicare beneficiaries (73.6 years in non-completers vs. 72.0 years in analytic sample), Medicaid beneficiaries (46.4 years in non-completers vs. 48.3 years in analytic sample), Private beneficiaries (44.3 years in non-completers vs. 49.6 years in analytic sample). The majority of Medicare and Medicaid beneficiaries were female in both samples. 55% of Private insurers were male among Private beneficiaries in non-completers vs. 54% female for Private beneficiaries in the analytic sample. Similar to the analytic sample, majority of Medicare beneficiaries were non-Hispanic White (61% in non-completers vs. 75% in analytic sample). Majority of Medicaid beneficiaries were Hispanic and non-Hispanic Black (59% in non-completers vs. 53% in analytic sample), and non-Hispanic White in Private beneficiaries (55% in non-completers vs. 65% in analytic sample). Family income level was also similarly distributed. Majority of private beneficiaries were high income (51% in non-completers vs. 54% in analytic sample). Majority of Medicaid beneficiaries were poor and near poor (53% in non-completers vs. 58% in analytic sample). Majority of Medicare beneficiaries were middle and high income (52% in non-completers vs. 62% in analytic sample). U.S. Census region was similarly distributed across insurance groups among non-completers and analytic sample.

© 2024 Mohan G et al. *JAMA Network Open.*

eTable 2. Sample Before Complete Case Analysis (N=16 244)

	Medicare (N=2,317)	Medicaid (N=3,092)	Private (N=10,835)	Overall (N=16,244)
**Age**				
Mean (SD), y	72.4 (7.9)	49.0 (18.2)	49.8 (17.2)	52.8 (18.2)
**Gender**				
Male	919 (39.7%)	1119 (36.2%)	4992 (46.1%)	7038 (43.3%)
Female	1398 (60.3%)	1973 (63.8%)	5843 (53.9%)	9214 (56.7%)
**Race and ethnicity**				
Hispanic	229 (9.9%)	941 (30.4%)	1637 (15.1%)	2807 (17.3%)
Non-Hispanic Asian	65 (2.8%)	104 (3.4%)	710 (6.6%)	879 (5.4%)
Non-Hispanic Black	265 (11.4%)	738 (23.9%)	1209 (11.2%)	2212 (13.6%)
Non-Hispanic White	1709 (73.8%)	1190 (38.5%)	6981 (64.4%)	9880 (60.8%)
Non-Hispanic Other	49 (2.1%)	119 (3.9%)	298 (2.8%)	466 (2.9%)
**Family income level**				
Poor (<100%)	330 (14.2%)	1456 (47.1%)	547 (5.1%)	2333 (14.4%)
Near Poor (100-124%)	162 (7.0%)	361 (11.7%)	246 (2.3%)	769 (4.7%)
Low (125-199%)	427 (18.4%)	552 (17.9%)	1001 (9.2%)	1980 (12.2%)
Middle (200-399%)	685 (30.0%)	545 (17.6%)	3267 (30.2%)	4497 (27.7%)
High (≥400%)	713 (30.8%)	178 (5.8%)	5774 (53.3%)	6665 (41.0%)
**U.S. Census Region**				
Northeast	375 (16.4%)	518 (16.9%)	1742 (16.1%)	2635 (16.3%)
Midwest	466 (20.4%)	545 (17.7%)	2374 (22.0%)	3385 (20.9%)
South	852 (37.2%)	1105 (36.0%)	3835 (35.5%)	5792 (35.8%)
West	595 (26.0%)	905 (29.5%)	2854 (26.4%)	4354 (26.9%)

To examine potential selection bias from complete case analysis, we examined the demographic characteristics of sample before complete case analysis and after (our analytic sample). The demographic characteristics of the participants in the sample before complete case analysis was performed (N= 16,244) were similar overall to complete cases in our analytic sample (N = 14,918), indicating minimal risk for selection bias from missingness. The mean age was similar in both samples across the three insurance groups: Medicare beneficiaries (72.4 years

© 2024 Mohan G et al. *JAMA Network Open.*

vs. 72.0 years in analytic sample), Medicaid beneficiaries (49.0 years vs. 48.3 years in analytic sample), Private beneficiaries (49.8 years vs. 49.6 years in analytic sample). The distribution for age was across the three insurance groups were similar in both samples. The majority of participants were females in both samples (56.7% vs. 56.8% in complete cases) and consistent across Medicare, Medicaid, and Private beneficiaries both samples. For race/ethnicity, the majority of Medicaid beneficiaries were Hispanic and non-Hispanic Black (54.3% vs. 53.3% in complete cases). The majority of Medicare beneficiaries (73.8% vs. 75.0% in complete cases) and Private beneficiaries (64.4% vs. 65.1% in complete cases) were non-Hispanic White. For family income level, the proportion of Medicaid beneficiaries with poor income was 47.1% vs. 46.2% in complete cases. The proportion of Medicare beneficiaries with middle and high income was 60.8% vs. 62.8% in complete cases. The proportion of private beneficiaries with high income was 53.3% vs. 54.2% in analytic sample. U.S. Census region was similarly distributed across insurance groups among non-completers and analytic sample.

© 2024 Mohan G et al. *JAMA Network Open.*

eTable 3. Pooled Model Joint Significance Testing

**Exposures**	**F-statistic**	**p value**
Educational Attainment	2.91	<0.001
Access to Medical Care	1.76	0.025
Medical Discrimination	3.82	<0.001
Housing Affordability	2.69	<0.001
Access to Healthy Foods	2.26	0.002
Availability of Parks	3.56	<0.001
Contacted by Collections in the past 12 months	3.37	0.001
Ability to cover unexpected expenses	3.29	0.001
Feeling socially isolated	2.89	<0.001
Attending club/organization meetings per year	3.39	<0.001
**Joint Adjusted Wald Test**	**3.59**	**<0.001**

We fit a pooled regression model, combining observations from all three insurance groups (e.g., Medicare, Medicaid, and private). This model included interaction terms between the insurance group and each of our exposure variables of interest. An adjusted Wald test was then performed to test for significance of each interaction term and then joint significance of all the interaction terms. We found there to be significant differences in the association between exposures and insurer expenditures across the insurance groups (p<0.001), justifying our initial approach for stratifying our analysis by payer.

© 2024 Mohan G et al. *JAMA Network Open.*

eTable 4. Variance Inflation Test (VIF) Results

**Variable**	**VIF**	**1/VIF**
Age	1.08	0.924197
Gender	1.03	0.971618
Race/Ethnicity	1.02	0.976866
U.S. Census Region	1.03	0.970135
Dual Coverage	1.18	0.848400
Educational Attainment	1.34	0.746314
Access to Medical Care	1.98	0.504525
Medical Discrimination	1.07	0.931339
Housing Affordability	1.24	0.809544
Access to Healthy Foods	2.06	0.486076
Availability of Parks	1.90	0.527218
Contacted by Collections in the past 12 months	1.16	0.863973
Ability to cover unexpected expenses	1.35	0.738374
Feeling socially isolated	1.11	0.903735
Attending club/organization meetings per year	1.08	0.922876

Mean VIF = 1.31

Threshold for concern: VIF

VIF<10 indicates full rank or no perfect collinearity

© 2024 Mohan G et al. *JAMA Network Open.*

eTable 5. Breusch-Pagan Test for Heteroskedasticity Results

**Fitted Values**	**X^2 test statistic**	**p-value**
Medicaid Expenditures	6433.30	<0.001
Medicare Expenditures	10965.51	<0.001
Private Expenditures	3464.57	<0.001

Assumption: Normal error terms

H_0: Constant Variance

© 2024 Mohan G et al. *JAMA Network Open.*

eTable 6. Park Test Results

Fitted Values	Coefficient (95% CI)	Standard Error	t-statistic	p-value
Slope	1.988 (1.832, 2.142)	0.079	25.25	<0.001
Intercept	-1.674 (-2.019, -1.329)	0.175	-9.55	<0.001

A Park test was performed by regressing the log of square residuals against the log of fitted values from a preliminary linear model. A slope coefficient close to 0 representing constant variance would indicate Gaussian family, coefficient close to 1 representing variance proportional to the mean would indicate a Poisson family, and coefficient close to 2 representing variance proportional to the square of the mean would indicate a Gamma family.

© 2024 Mohan G et al. *JAMA Network Open.*
