# Supplementary material for: Social Determinants of Health and US Health Care Expenditures by Insurer
Source: JAMA Netw Open. 2024 Oct 23;7(10):e2440467. doi: 10.1001/jamanetworkopen.2024.40467 (PMC11581502; doi:10.1001/jamanetworkopen.2024.40467)
Supplement: Supplement 2. — Data Sharing Statement [file jamanetwopen-e2440467-s002.pdf]

Data Sharing Statement

Mohan. Social Determinants of Health and US Health Care Expenditures by Insurer. *JAMA Netw Open*. Published October 23, 2024. doi:10.1001/jamanetworkopen.2024.40467

Data
Data available: Yes
Data types: Deidentified participant data
How to access data: https://meps.ahrq.gov/data_stats/download_data_files.jsp
When available: With publication

Supporting Documents
Document types: None

Additional Information
Who can access the data: Anyone requesting the data
Types of analyses: For any purpose
Mechanisms of data availability: With or without investigator support
